# Overexpression of CD44 Variant 9: A Novel Cancer Stem Cell Marker in Human Cholangiocarcinoma in Relation to Inflammation

**DOI:** 10.1155/2018/4867234

**Published:** 2018-10-09

**Authors:** Nattawan Suwannakul, Ning Ma, Raynoo Thanan, Somchai Pinlaor, Piti Ungarreevittaya, Kaoru Midorikawa, Yusuke Hiraku, Shinji Oikawa, Shosuke Kawanishi, Mariko Murata

**Affiliations:** ^1^Department of Environmental and Molecular Medicine, Mie University Graduate School of Medicine, Tsu, Mie 514-8507, Japan; ^2^Graduate School of Health Science, Suzuka University of Medical Science, Suzuka, Mie 513-8670, Japan; ^3^Department of Biochemistry, Faculty of Medicine, Khon Kaen University, Khon Kaen 40002, Thailand; ^4^Cholangiocarcinoma Research Institute, Khon Kaen University, Khon Kaen 40002, Thailand; ^5^Department of Parasitology, Faculty of Medicine, Khon Kaen University, Khon Kaen 40002, Thailand; ^6^Department of Pathology, Faculty of Medicine, Khon Kaen University, Khon Kaen 40002, Thailand; ^7^Faculty of Pharmaceutical Sciences, Suzuka University of Medical Science, Suzuka, Mie 513-8670, Japan

## Abstract

Various CD44 isoforms are expressed in several cancer stem cells during tumor progression and metastasis. In particular, CD44 variant 9 (CD44v9) is highly expressed in chronic inflammation-induced cancer. We investigated the expression of CD44v9 and assessed whether CD44v9 is a selective biomarker of human cholangiocarcinoma (CCA). The expression profile of CD44v9 was evaluated in human liver fluke *Opisthorchis viverrini*-related CCA (OV-CCA) tissues, human CCA (independent of OV infection, non-OV-CCA) tissues, and normal liver tissues. CD44v9 overexpression was detected by immunohistochemistry (IHC) in CCA tissues. There was a higher level of CD44v9 expression and IHC score in OV-CCA tissues than in non-OV-CCA tissues, and there was no CD44v9 staining in the bile duct cells of normal liver tissues. In addition, we observed significantly higher expression of inflammation-related markers, such as S100P and COX-2, in OV-CCA tissues compared to that in non-OV and normal liver tissues. Thus, these findings suggest that CD44v9 may be a novel candidate CCA stem cell marker and may be related to inflammation-associated cancer development.

## 1. Introduction

Infection and chronic inflammation are important factors for carcinogenesis, and cholangiocarcinoma (CCA) is a specific type of inflammation-associated cancer. Potential risk factors of CCA are parasitic infections (*Opisthorchis viverrini* and *Clonorchis sinensis*), bile duct disorders (biliary tract cysts and hepatolithiasis), toxins, complications (diabetes, cirrhosis, and obesity), alcohol consumption, and smoking [1]. The incidence of CCA has risen globally, and the highest rate occurs in Thailand, particularly in northeastern regions such as Khon Kaen. In Khon Kaen Province, high prevalence of CCA cases is related to *Opisthorchis viverrini* (OV) infection [2]. OV infection increases inflammation and enlarges bile ducts and connective tissues, resulting in periductal fibrosis and eventually the development of bile duct cancer [3]. The diagnosis of CCA is difficult because of clinical silence and a nonspecific appearance. Discovering specific molecular biomarkers may aid early and definitive diagnosis of CCA.

CD44 is a transmembrane glycoprotein that is ubiquitously synthesized and expressed in several types of mammalian cells such as leukocytes, red blood cells, brain cells, and epithelial cells [4]. Alternative splicing results in several isoforms of CD44 with different functions. The standard isoform of CD44 (CD44s) is generally expressed in most normal epithelial cells, and variant isoforms of CD44 (CD44v) are expressed in some epithelial-type carcinomas [5]. CD44v is involved in cell proliferation, differentiation, migration, and adhesion in normal cells and metastasis in cancer cells. Recently, CD44v was proposed as a stem cell marker for several types of cancer [6]. In particular, CD44 variant 9 (CD44v9) is overexpressed in bladder cancer, pancreatic cancer, and colon cancer [7–9]. Overexpression of CD44v9 in gastric cancer caused by chronic inflammation from *Helicobacter pylori* infection is a prognostic biomarker at an early stage and is a predictive marker for recurrence [10, 11]. These findings suggest that CD44v9 is a selective target for inflammation-related CCA.

In this study, we examined the expression of CD44v9 in OV-related CCA (OV-CCA) tissues in comparison with that in normal bile duct cells and tissues of CCA independent of OV infection (non-OV-CCA) by immunohistochemistry (IHC). S100 calcium-binding protein P (S100P) is an important mediator of cancer-related inflammation [12–14], which leads to tumor invasion and metastasis [15], and S100P was identified as a CCA biomarker in both non-OV-CCA [16] and OV-CCA [17]. Overexpression of S100P may help to predict the clinical outcome of CCA patients [18, 19]. Cyclooxygenase-2 (COX-2) is also involved in inflammation, and we analyzed both COX-2 and S100P to clarify the relationship between OV-related CCA and inflammation.

## 2. Materials and Methods

### 2.1. Tissue Samples and Clinical Data

Thirty-three human liver fluke-caused CCA tissues (26 males, 7 females) were obtained from the Cholangiocarcinoma Research Institute of Srinagarind Hospital, Khon Kaen University, Khon Kaen, Thailand. The collection of CCA tissues was approved by the Ethics Committee for Human Research (HE571283), Khon Kaen University, Thailand. Informed consent was obtained from all CCA patients. Clinical data of patients were recorded including age and sex. All cancer tissues were classified using AJCC 7th edition of TMN staging [20].

Human tissue microarray slides including normal liver tissues (21 cases: 12 males, 9 females) and CCA tissues (98 cases: 56 males, 42 females) were purchased from US Biomax Inc. (LVN801 and LV1004, Derwood, Maryland, USA). CCA tissues were classified in various TNM classes and tumor stages.

### 2.2. Immunohistochemical Staining (IHC) and Scoring

Embedded tissue sections were deparaffinized by xylene and rehydrated using a graded series of ethanol solutions. Antigen retrieval was performed by heating tissue sections in 5% urea at 500 watts in a microwave oven for 5 minutes. Endogenous peroxidase activity was quenched with 1% H_2_O_2_ (Kanto Chemical, Tokyo, Japan) followed by blocking with 1% skim milk (BD Biosciences, San Jose, California, USA). The sections were treated with primary antibodies (rat anti-CD44v9 monoclonal antibody from Cosmo Bio, Tokyo, Japan; goat anti-COX-2 from Santa Cruz Biotechnology, Dallas, Texas, USA; and rabbit anti-S100P from Abcam Biotechnology, Cambridge, UK, diluted 1 : 300 each) in a humid chamber. The sections were then incubated with biotinylated secondary antibody (Vector Laboratories, Burlingame, California, USA) including rabbit anti-rat IgG, rabbit anti-goat IgG, and goat anti-rabbit IgG. All sections were incubated with an avidin-biotin-peroxidase conjugate. The immunoreaction was activated by a peroxidase stain DAB kit (Nacalai Tesque, Kyoto, Japan), counterstained with hematoxylin, and mounted with Entellan New (Merck Millipore, Darmstadt, Germany). Stained tissues were visualized under a microscope (Olympus BX53F, Tokyo, Japan). The intensity of staining was graded by an IHC score between 0 and 4 by two investigators as follows: no staining (0), weak staining (1+), moderate staining (2+), strong staining (3+), and very strong staining (4+) in cholangiocytes and CCA cancer cells. The representative images for each score are shown in Figure S1A.

Paraffin-embedded OV-CCA tissues from 18 patients were stained using a double fluorescent staining method. Primary antibodies (anti-CD44v9, S100P, and COX-2 antibodies) were used at a dilution of 1 : 200, and secondary antibodies (donkey-anti-rat IgG Alexa fluor 488 and 594, donkey-anti-rabbit IgG Alexa fluor 488, and donkey-anti-goat IgG Alexa fluor 594) (Abcam Biotechnology, Cambridge, UK) were used at a dilution of 1 : 400. Nuclei were stained using DAPI (Southern Biotech, Birmingham, Alabama, USA). Stained tissues were examined by a fluorescent microscope (Olympus BX53F, Tokyo, Japan).

### 2.3. Statistical Analysis

Statistical analysis was performed using an SPSS software version 23.0 (IBM Corporation, USA). To compare patient clinical data between three groups, a chi-square test was used for age distribution, sex ratio, and TNM stage. The nonparametric Kruskal-Wallis test was performed to determine significant differences in IHC scores between the three groups, followed by multiple comparison with an adjustment of *p* value by the Bonferroni method (a pairwise test smaller than 0.05/3 = 0.017 was significant at the 0.05 level and 0.01/3 = 0.0033 at the 0.01 level). Statistical analysis was considered significant at *p* < 0.05.

## 3. Results

### 3.1. Clinical Features of CCA Patients

The summary of sample data is shown in Table 1. The age distribution was younger in the subjects of normal liver tissue array samples than those of non-OV and OV-CCA patients. There was no significant difference in age distribution between non-OV and OV-CCA groups. The sex ratio was not significantly different among the three groups. Patients with OV-CCA had more severe cancer progression than non-OV-CCA patients and had a significantly higher degree of tumor grade (T; T1–2 vs. T3–4), lymph node metastasis grade (N; N0 vs. N1), and distant metastasis grade (M; M0 vs. M1). There was an increased number of higher stages (III + IV) in OV-CCA cases than in non-OV-CCA cases.

### 3.2. Overexpression of CD44v9 in Human CCA Tissues

**Figure 1:**
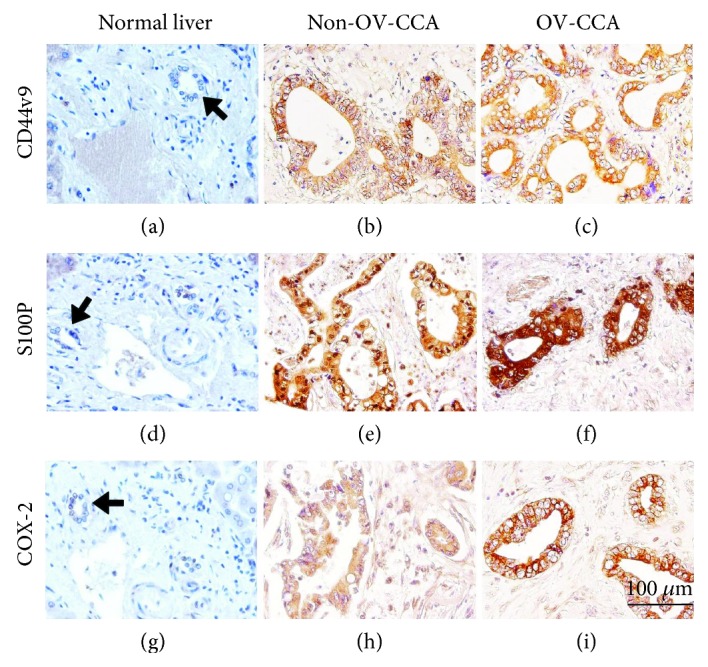
Immunohistochemical staining for CD44v9 (a–c), S100P (d–f), and COX-2 (g–i) in human liver tissues. Normal liver (a, d, g), non-OV-CCA (b, e, h), and OV-CCA tissues (c, f, i). Arrows indicate normal cholangiocytes.

**Figure 2:**
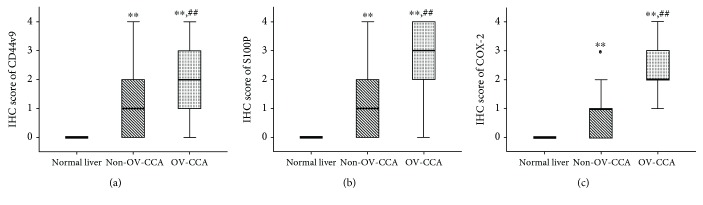
Box plots of CD44v9 (a), S100P (b), and COX-2 (c) staining in human liver tissues categorized by IHC score. The horizontal bold lines represent the median value, and the lower and upper boxes represent the 25th and 75th percentiles, respectively. The whiskers represent the range of data, and the circle is an outlier. A Kruskal-Wallis test was used to test for a significance difference among the three groups, and a Mann-Whitney *U* test was used to compare two groups with an adjustment of *p* value by the Bonferroni method. ^∗∗^ *p* < 0.01 compared to the normal liver group and ##*p* < 0.01 compared to the non-OV-CCA group.

Cholangiocytes in normal liver tissues had no CD44v9 staining by IHC (Figure 1(a)). In CCA tissues, CD44v9 expression was observed in the membrane and cytoplasm of cancer cells (Figures 1(b) and 1(c)). There were no stains in these tissues when the primary antibodies were omitted (Figure S1B). In normal liver samples (*n* = 21), there was no positive CD44v9 staining (0%). CD44v9 was stained in 55.1% of non-OV-CCA (*n* = 98) and 87.9% of OV-CCA cases (*n* = 33). The IHC score of CD44v9 was significantly higher in CCA tissues than in normal tissues (Figure 2(a)). Furthermore, the CD44v9 staining score was significantly higher in the OV-CCA group than in the non-OV-CCA group.

### 3.3. Expression of Inflammation-Related Markers in CCA Tissues

S100P staining was not detected in bile duct cells of normal liver tissues (Figure 1(d)). In contrast, S100P staining occurred in the nucleus and cytoplasm of cancer cells in CCA tissues (Figures 1(e) and 1(f)). Without the primary antibody, no staining was observed in these tissues (Figure S1B). All normal liver tissue samples were negatively stained. S100P expression was observed in 58.2% of non-OV-CCA cases and 97.0% of OV-CCA cases. CCA tissues had markedly greater IHC scores than normal liver tissues, and scores were significantly higher in OV-CCA tissues than in non-OV-CCA tissues (Figure 2(b)).

Similarly, COX-2 expression was not observed in cholangiocytes of normal liver tissues (Figure 1(g)). COX-2 was overexpressed in the cytoplasm and nucleus of bile duct cancer cells (Figures 1(h) and 1(i)). The stained tissues were not detected by excluding primary antibodies (Figure S1B). COX-2 staining was positive in 56.1% of non-OV tissues and 100% of OV-CCA tissues. COX-2 IHC scores were significantly higher in both non-OV and OV-CCA tissues compared to that in normal liver tissues, and the scores in OV-CCA tissues were significantly higher than that in non-OV tissues (Figure 2(c)).

Among the expression of these molecules in non-OV-CCA and OV-CCA samples, there are significant correlations between the staining intensities of CD44v9 and S100P or COX-2 by Spearman's rank correlation coefficient (*r*). The correlations were moderate between CD44v9 and S100P (*r* = 0.455, *p* < 0.001) and also CD44v9 and COX-2 (*r* = 0.465, *p* < 0.001).

### 3.4. Double Fluorescent Staining for Human OV-CCA Tissues

**Figure 3:**
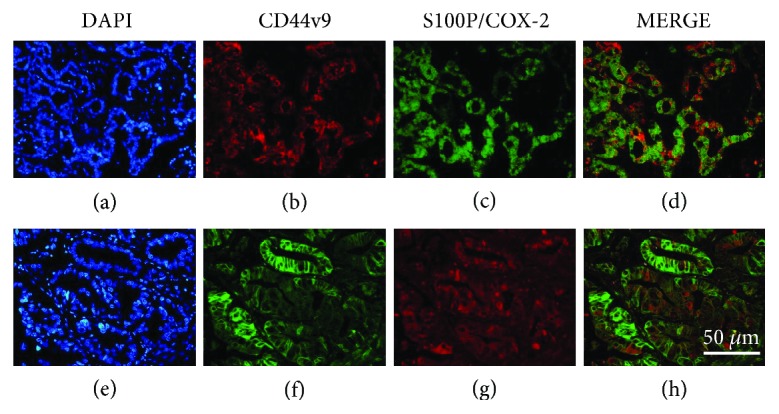
Representative images of double fluorescent staining in human OV-CCA tissues. DAPI staining of nuclei (a, e), CD44v9 (b, f), S100P (c), COX-2 (g), and merge (d, h).

In OV-CCA tissues, CD44v9 was primarily expressed in the cell membrane and moderately in the cytoplasm of CCA cells (Figures 3(b) and 3(f)), and S100P mostly appeared in the cytoplasm and nucleus of cancer cells (Figure 3(c)). Double-positive cells for CD44v9 and S100P were observed in OV-CCA tissues (Figure 3(d)). COX-2 was expressed in the nucleus and cytoplasm of cancer cells (Figure 3(g)). Both CD44v9 and COX-2 were expressed in some cancer cells of OV-CCA tissues (Figure 3(h)).

## 4. Discussion

We firstly evaluated CD44v9 as a candidate biomarker of OV-related CCA by IHC. The level of CD44v9 expression was significantly higher in cancer cells of CCA patients, particularly in OV-CCA, than in normal liver tissues. We also investigated the expression of inflammatory markers, S100P and COX-2, which were overexpressed in CCA tissues, predominantly in OV-CCA.

The alternative splicing of human CD44 gene produces different CD44 variant isoforms, which are abundantly expressed in several tumors, while the standard isoform is mainly expressed in normal epithelial cells [21]. In multiple complexes of variant isoforms, the exon combination of variable regions provides a heterogeneity of CD44 molecules. In exon combination, isoforms of CD44 include a property of tissue-specific expression by distinct expression in different tissues with normal or diseased states [22]. These combinations predominantly are exhibited in specialized tissues, e.g., CD44v3-10 is a keratinocyte form and CD44v8-10 is an epithelial form [23–25]. Moreover, the various isoforms of CD44 could present a broad spectrum of physiological functions and may have defined functions [26, 27] depending on a number of exon combinations through its posttranslational modification of CD44 splicing variant, which impacted on the molecular interactions and tumorigenicity [28]. Non-Hodgkin's lymphoma (NHL) showed a different expression pattern between individual v6 and v6-containing isoforms. A single CD44v6 exon composition was expressed in low-grade NHL, while the combination of v6 exon with other variant exons was presented in high-grade NHL [29]. Different tissues express different CD44 variants with various exon combinations, and the cellular functions might relate to its variable of exon combination. Among various exon combinations, CD44v9 is involved in several exon combinations such as v1-10, v6-10, v7-10, and v8-10. In gastric adenocarcinoma, CD44v9 is contained in v6-10, v7-10, and v8-10 combinations [30]. CD44v8-10 has a role of cancer stem cell in CCA development via redox regulation [31]. Defects in mRNA splicing are an important cause of cancer, and the most common form of splicing defects are genomic splice site point mutations [32]. The present study showed the existence of CD44v9 in CCA, and our previous studies [33, 34] indicated inflammation-related DNA damage. Further study is needed to clarify the molecular mechanisms of CCA development mediated by splicing defects.

Inflammation is a fundamental cancer-promoting factor. OV infection causes the production of reactive oxygen species (ROS), such as nitric oxide and superoxide, which leads to the formation of DNA lesions, including 8-nitroguanine and 8-oxo-7,8-dihydro-2′-deoxyguanosine (8-oxodG) [33, 34], indicating that OV infection is a cause of inflammation-associated carcinogenesis. Previously, we found a high level of expression of stem cell markers, CD133 and Oct3/4, and a high level of 8-oxodG in OV-CCA tissues, suggesting that stem cell mutations are involved in the inflammatory microenvironment during CCA development [35]. CD44 variant isoforms including CD44v9 mitigate ROS [31, 36], which may explain the high level of expression of CD44v9 in addition to S100P and COX-2 predominantly in the tissues of OV-CCA, a cancer driven by inflammation. CD44v9-positive cells were detected in both non-OV and OV-CCA tissues. Some non-OV-CCA tissues expressed CD44v9, which may be explained by a report that solid tumors including CCA are under inflammatory conditions including hypoxia [37]. Additionally, a correlation between the expression of S100P and receptor for advanced glycation end product (RAGE) has been reported [38] and might be involved in an inflammatory response including COX-2 induction. We postulated environmental factor-related inflammation such as OV infection and tumor-producing inflammation such as hypoxia and S100P-RAGE in the multiple steps of carcinogenesis [39]. OV-CCA may be affected by both OV infection and tumor-producing inflammation, and non-OV-CCA may be affected by tumor-producing inflammation alone. Interestingly, in this study, significant positive correlations were observed between the staining of CD44v9 and S100P or COX-2, suggesting a role of CD44v9 in inflammation. This finding raises a possibility that CD44v9 expression is associated with an inflammatory state during CCA development.

Previously, we found that prolonged oxidative stress induces stem cell properties via gene downregulation, which results in CCA genesis with aggressive clinical outcomes [40]. Qu et al. observed the coexpression of CD44 and S100P protein in spheroid-forming pancreatic ductal epithelial cells chronically treated with cadmium, which suggests that S100P expression likely contributes to the aggressive and stemness nature of spheroids [41]. In an inflammatory microenvironment, COX-2 stimulates the development of breast cancer stem cells [42], and the proliferation of CD44^+^ stem-like cells in gastric cancer is cooperatively stimulated by COX-2/PGE2-mediated signaling [43]. Previously, we found that COX-2 activation may be involved in inflammation-mediated stem cell proliferation and differentiation in urinary bladder carcinogenesis [44]. Although it is still unclear how inflammation affects cancer cell stemness, our results suggest a positive correlation between CD44v9 and inflammation.

Several studies found that CD44v9 expression is associated with cancer tumorigenicity. Kiuchi et al. observed a high expression of CD44v9 in the progression of pancreatic cancer cells during mitosis [8]. Additionally, CD44v9-expressed cells have apoptotic resistance and enhanced invasive properties [45, 46]. Wang et al. illustrated that a small population of CD44-positive CCA cells have properties of cancer stem cells including self-renewal [47]. Seishima et al. reported that the anti-inflammatory drug sulfasalazine reduces proliferation of CD44v9-positive cells and has a significant impact on ulcerative colitis-associated tumor cell differentiation [48]. Similarly, the formation of cancer cells in inflammation-mediated human gastric adenocarcinoma is correlated with CD44v9 expression. In addition, CD44v9-ablated or sulfasalazine-treated mice have reduced expansion of gastric tumor cells and development of premalignant lesions in the stomach [49]. We observed overexpression of CD44v9 in CCA, especially in OV-CCA. Taken together, these data indicate the importance of CD44v9-positive cancer stem cells in the progression of inflammation-related cancer. Reassessment of anti-inflammatory drugs may be a valuable approach to develop new chemotherapies. Further investigation of CD44v9-targeted cancer stem cells is needed, and the overexpression of CD44v9 in CCA may point to new anticancer stem cell therapeutic strategies.

## Figures and Tables

**Table 1:** Clinicopathological information of normal subjects and CCA patients.

Characteristics	Normal liver *n* = 21 No. (%)	Non-OV-CCA *n* = 98 No. (%)	OV-CCA *n* = 33 No. (%)	Statistical significance
Age (years)				
≤40	15 (71.4)	12 (12.2)	2 (6.1)	^∗∗^
>40	6 (28.6)	86 (87.8)	31 (93.9)	
Sex				
Male	12 (57.1)	56 (57.1)	26 (78.8)	n.s.
Female	9 (42.9)	42 (42.9)	7 (21.2)	
TNM classification				
T^a^				
T1	—	2 (2.0)	1 (3.7)	^##^
T2	—	51 (52.0)	5 (18.5)	
T3	—	43 (43.9)	9 (33.3)	
T4	—	2 (2.0)	12 (44.4)	
N^a^	—			
N0	—	67 (68.4)	3 (20.0)	^##^
N1	—	31 (31.6)	12 (80.0)	
M^a^	—			
M0	—	95 (96.9)	24 (85.7)	^#^
M1	—	3 (3.1)	4 (14.3)	
Tumor stage^a^				
I	—	2 (2.0)	0 (0.0)	^##^
II	—	40 (40.8)	3 (11.5)	
III	—	27 (27.6)	7 (26.9)	
IV	—	29 (29.6)	16 (61.6)	

OV: *Opisthorchis viverrini*; CCA: cholangiocarcinoma; n.s.: not significant. ^∗∗^ *p* < 0.01 compared to that of the normal liver group. ^#^ *p* < 0.05 and ^##^ *p* < 0.01 compared to that of the non-OV-CCA group. ^a^Patients missing clinical information are not included in the statistical analyses.

## Data Availability

The data used to support the findings of this study are available from the corresponding author upon request.

## Supplementary Materials

Supplementary Materials Supplementary Figure S1: IHC scoring and negative control. (A) Representative CCA examples of negative and positive staining of CD44v9 (A–E), S100P (F–J), and COX-2 (K–O) by scoring range as follows: 0 for no staining (A, F, K), 1+ for weak staining (B, G, L), 2+ for moderate staining (C, H, M), 3+ for strong staining (D, I, N), and 4+ for very strong staining (E, J, O). (B) Representative CCA examples of tissue staining without primary antibodies. CCA tissues were stained with only secondary antibodies (VECTASTAIN Elite ABC HRP Kit), including rabbit anti-rat IgG for CD44v9 (PK-6104), goat anti-rabbit IgG for S100P (PK-6101), and rabbit anti-goat IgG for COX-2 (PK-6105).
