# Supplementary material for: Overexpression of CD44 Variant 9: A Novel Cancer Stem Cell Marker in Human Cholangiocarcinoma in Relation to Inflammation
Source: Mediators Inflamm. 2018 Oct 9;2018:4867234. doi: 10.1155/2018/4867234 (PMC6198546; doi:10.1155/2018/4867234)
Supplement: Supplementary Materials — Supplementary Figure S1: IHC scoring and negative control. (A) Representative CCA examples of negative and positive staining of CD44v9 (A–E), S100P (F–J), and COX-2 (K–O) by scoring range as follows: 0 for no staining (A, F, K), 1+ for weak staining (B, G, L), 2+ for moderate staining (C, H, M), 3+ for strong staining (D, I, N), and 4+ for very strong staining (E, J, O). (B) Representative CCA examples of tissue staining without primary antibodies. CCA tissues were stained with only secondary antibodies (VECTASTAIN Elite ABC HRP Kit), including rabbit anti-rat IgG for CD44v9 (PK-6104), goat anti-rabbit IgG for S100P (PK-6101), and rabbit anti-goat IgG for COX-2 (PK-6105). [file 4867234.f1.docx]

**Supplementary Figure S1.**


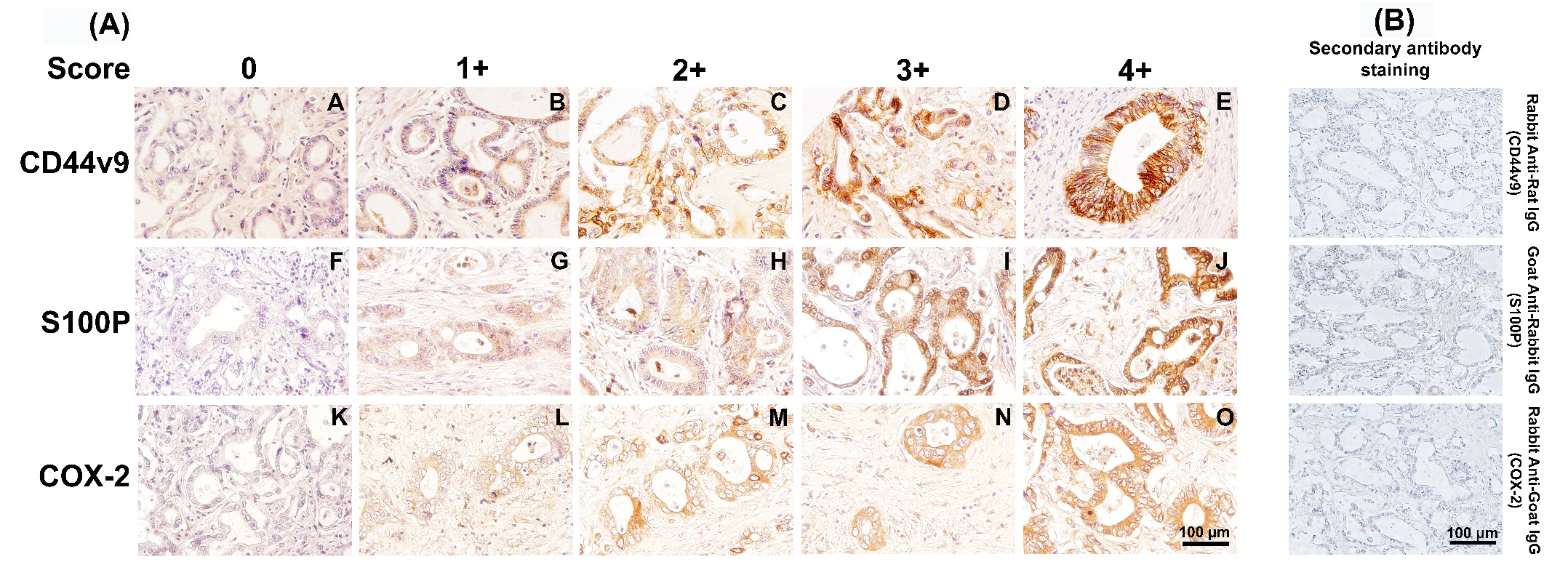


**Supplementary Figure S1**. **IHC scoring and negative control.** **(A)** Representative CCA examples of negative and positive staining of CD44v9 (A-E), S100P (F-J) and COX-2 (K-O) by scoring range as follow: 0 for no staining (A, F, K), 1+ for weak staining (B, G, L), 2+ for moderate staining (C, H, M), 3+ for strong staining (D, I, N), 4+ for very strong staining (E, J, O). **(B)** Representative CCA examples of tissue staining without primary antibodies. CCA tissues were stained with only secondary antibodies (VECTASTAIN Elite ABC HRP Kit), including rabbit anti-rat IgG for CD44v9 (PK-6104), goat anti-rabbit IgG for S100P (PK-6101) and rabbit anti-goat IgG for COX-2 (PK-6105).
